# Elucidating the role of Pyroptosis in papillary thyroid cancer: prognostic, immunological, and therapeutic perspectives

**DOI:** 10.1186/s12935-024-03229-0

**Published:** 2024-01-29

**Authors:** Fang Li, Rui Du, Jiedong Kou, Jingting Li, Le Zhou, Daqi Zhang, Yantao Fu, Gianlorenzo Dionigi, Simona Bertoli, Hui Sun, Nan Liang

**Affiliations:** 1 Division of Thyroid Surgery, The China-Japan Union Hospital of Jilin University, Jilin Provincial Key Laboratory of Surgical Translational Medicine, Jilin Provincial Precision Medicine Laboratory of Molecular Biology and Translational Medicine on Differentiated Thyroid Carcinoma, Changchun, 130031 Jilin China; 2 Division of General Surgery, Endocrine Surgery Unit, Endocrine Surgery Research Institute, Istituto Auxologico Italiano Capitanio (IRCCS), University of Milan, 20122 Milan, Italy; 3 Department of Food, Environmental and Nutritional Sciences (DeFENS), University of Milan, 20133 Milan, Italy

**Keywords:** Pyroptosis, Thyroid cancer, Tumor immune microenvironment, Prognosis, Therapy

## Abstract

**Background:**

Pyroptosis, an inflammatory form of programmed cell death, has been implicated in the pathogenesis and progression of several cancers. However, the significance of pyroptosis-related genes (PRGs) in papillary thyroid cancer (PTC) remains unclear.

**Methods:**

Transcriptome and clinical data of PTC patients were obtained from The Cancer Genome Atlas. The expression patterns of PRGs were identified by consensus clustering. A prognostic model for predicting the thyroid cancer-free interval (TCFi) employed five machine learning methods. Enrichment and immune-related analyses were performed to elucidate the role of pyroptosis. The responses to radioactive iodine (RAI), immune checkpoint inhibitors (ICIs), molecular targeted therapy (MTT), and chemotherapy (CTx) were predicted based on pyroptosis-derived features. Additionally, the expression of prognostic PRGs was validated via six external datasets, 16 cell lines, and 20 pairs of clinical samples.

**Results:**

PTC patients were classified into three PyroClusters, C1 exhibited BRFA-like tumors with the highest invasiveness and the worst prognosis, C2 presented RAS-like tumors, and C3 was characterized by gene fusion. Nine PRGs (CXCL8, GJA1, H2BC8, IFI27, PRDM1, PYCARD, SEZ6L2, SIGLEC15, TRAF6) were filtered out to construct a PyroScore prognostic model. A derived nomogram demonstrated superior predictive performance than four clinical staging systems. A strong correlation between pyroptosis and tumor immune microenvironment (TIME) remodeling was observed in mechanistic analyses. Patients with a high PyroScore exhibited “hot” tumor immunophenotypes and had a poorer prognosis but could benefit more from ICIs and CTx (such as paclitaxel). Patients with a low PyroScore were more sensitive to RAI and MTT (such as pazopanib and sorafenib).

**Conclusions:**

PyroScore model can effectively predict TCFi in patients with PTC. Dysregulated expression of PRGs is associated with the TIME modeling. Pyroptosis features have potential significance for developing novel therapeutic strategies for PTC patients.

**Supplementary Information:**

The online version contains supplementary material available at 10.1186/s12935-024-03229-0.

## Introduction

Thyroid cancer (THCA) is the most prevalent endocrine malignancy, and its annual incidence rate continues to rise, papillary thyroid cancer (PTC) accounts for over 85% of THCA cases [1]. While most PTCs have a favorable prognosis with a five-year survival rate of 80%~95%, approximately 15%~40% of patients experience recurrence or progression after initial treatment. This not only complicates primary tumor management but also leads to a marked increase in the mortality rate (to over 60%) [2, 3]. Therefore, a key issue in managing PTC patients is to minimize the morbidity and mortality associated with recurrence and to find more effective treatment modalities to improve the prognosis.

Pyroptosis is a newly discovered form of programmed cell death characterized by the formation of membrane pores, subsequent cell swelling and lysis, and the release of proinflammatory substances [4]. Its role in various human diseases and its potential as therapeutic targets have been widely studied [5, 6]. Emerging evidence reveals that pyroptosis plays dual roles in cancer. On the one hand, pyroptosis within the tumor immune microenvironment (TIME) generates inflammasomes and cytokines that may promote tumorigenesis, immunosuppression, angiogenesis, and metastasis [5, 7]. On the other hand, pyroptosis triggers antitumor immunity by activating cytotoxic T cells, augmenting macrophage phagocytosis, and transforming “cold” tumors into “hot” tumors, thereby amplifying treatment-induced tumor cell death [6, 8]. This is similar to the dual role of thyroid autoimmune disease in the pathogenesis of THCA. Some studies suggest that chronic inflammation is a risk factor or precursor of PTC [9, 10], others indicate that strengthening the immune response may reduce tumor aggressiveness, leading to more favorable clinical outcomes [11, 12]. Accordingly, we speculated that pyroptosis, characterized by its proinflammatory nature, plays a role in the development of PTC. However, the molecular mechanisms and clinical implications of pyroptosis in PTC remain largely unknown.

Therefore, the aim of this study was to explore the role of pyroptosis in PTC and its potential applications in prognosis and therapy. Firstly, we investigated the expression patterns and clinical relevance of pyroptosis-related genes (PRGs) in PTC and then constructed a prognostic model for predicting the thyroid cancer-free interval (TCFi). Subsequently, we sought to further explore the mechanisms of pyroptosis involved in regulating the TIME. In addition, we would like to assess its potential applications in multimodal treatments for PTC.

## Materials and methods

### Data acquisition and preprocessing

Transcriptome profiles and clinical data of 572 THCA samples were downloaded from the TCGA database [13]. Tumor samples without complete prognostic information and those with less than four months of follow-up were excluded. Finally, 477 primary PTC and 59 normal thyroid (NT) samples were included in this study. Meanwhile, gene expression data were filtered to exclude genes with low expression whose average count value was < 100 across all samples. The raw count data were used for differential gene expression analysis between sample groups, while the DESeq2-normalized count data were used for downstream analysis. A total of 226 PRGs were derived from the MSigDB, the Reactome database, and the GeneCards (searching with the keyword ‘pyroptosis’ and screening with a relevance score above the median). Listed in Table S1.

### Definition of prognostically relevant events of PTC

Overall survival (OS) is minimal ambiguity in defining an event; the patient is either alive or dead. Progression-free survival (PFS) is generally considered a more informative endpoint for THCA studies; however, its events also include non-thyroid cancer specific events such as all-cause deaths and new tumor events. Specially, we defined the period from four months after initial treatment to events including recurrence, distant metastasis, biochemical evidence of disease and death with tumor as the thyroid cancer-free interval (TCFi) [14–16].

In the TCGA cohort, 308 PTC patients received radioactive iodine (RAI) treatment (PTC-RAI), and 169 patients did not undergo RAI. Combining the definition of RAI-refractory in the American Thyroid Association (ATA) guidelines with the limited information in the TCGA database, PTC-RAI patients were divided into a RAI-sensitive group (RS, *n* = 268) and a RAI-refractory group (RR, *n* = 40) according to whether TCFi events occurred after RAI treatment [17]. Furthermore, we acquired the GSE151179 cohort from the GEO database to validate the response to RAI treatment; this cohort included 4 patients in the RS group and 13 patients in the RR group [18].

### Identification of variants in pyroptosis phenotypes in PTC

Differentially expressed genes (DEGs) between PTC and NT tissues were identified using the package ‘DESeq2’. To explore the relationship between the pyroptosis-related DEGs and PTC subtypes, we then performed unsupervised consensus clustering via the k-means method using the package ‘ConsensusClusterPlus’. The correlations between the individual cluster and clinical characteristics were analyzed and presented using the packages ‘survival’, ‘survminer’ and ‘ComplexHeatmap’.

### Construction and validation of a PyroScore prognostic model

A total of 477 PTC patients were randomly split into training (*n* = 344) and testing (*n* = 143) sets at a ratio of 7:3 using the package ‘caret’ (Table S2). First, univariate Cox analysis was performed to assess the association between the expression of each PRG and the TCFi of patients in the training set. The cutoff value for the *P* value was set at 0.2 to avoid omissions. Then, random forest, least absolute shrinkage and selection operator regression, gradient boosting machine, decision tree, and extreme gradient boosting analyses were applied to rank the prognostic PRGs by importance using the ‘randomForest’, ‘glmnet’, ‘gbm’, ‘rpart’ and ‘xgboost’ packages. Next, multivariate Cox regression analysis was performed on the convergent PRGs for achieving coefficients, and the PyroScore was calculated using the predict() function.

The median score was taken as the cutoff value for stratifying patients into different PyroScore subgroups. Kaplan–Meier (KM) curve with log-rank test and receiver operating characteristic (ROC) curve analysis based on TCFi were performed using the ‘survminer’ and ‘timeROC’ packages. Principal component analysis (PCA) and t-distributed stochastic neighbor embedding (t-SNE) were used to visualize the separation of the PyroScore subgroups using the ‘Rtsne’ package. The prognostic value of the PyroScore was also verified in the testing set and the entire cohort by the same methods.

### Development and evaluation of the predictive performance of a nomogram

In the entire TCGA cohort, univariate and multivariate Cox regression analyses were applied to determine the independent prognostic value of the PyroScore. Subsequently, a nomogram integrating the PyroScore and clinicopathological factors was developed to investigate the probability of 1-, 3-, and 5-year TCFi using the ‘rms’ package. The time-dependent ROC, concordance index, calibration curves, and decision curve analysis (DCA) curves were used to assess the consistency, accuracy and net utility of the nomogram. Meanwhile, the availability of the nomogram was compared with that of the four THCA staging systems commonly used in clinical practice. These are the risk stratification system of ATA, the Metastases, Age, Completeness of Resection, Invasion, Size (MACIS), the American Joint Committee on Cancer (AJCC) 8th edition tumor, node, metastases (TNM) and the European Organization for Research and Treatment of Cancer (EORTC) [17, 19, 20].

### Functional enrichment analysis

Gene Ontology (GO) and Kyoto Encyclopedia of Genes and Genomes (KEGG) enrichment analyses were performed between PyroScore subgroups using the ‘clusterProfiler’ package. Gene set variation analysis (GSVA) was performed to determine the correlation with pyroptosis features and 50 hallmarks of cancer [21]. In addition, we compared the enrichment scores of 27 thyroid-related signatures via single sample gene set enrichment analysis (ssGSEA) using the ‘GSVA’ package, and the reference gene sets were derived from MSigDB and previous publications (Table S3).

### Assessment of immunological characteristics

The ‘estimate’ package was used to estimate the tumor microenvironment scores representing the proportion of immune and stromal cells and tumor purity. TIMER, XCELL, QUANTISEQ, MCPCOUNTER, EPIC, and CIBERSORT algorithms were applied to calculate the relative abundance of infiltrating immune cells. Meanwhile, the absolute enrichment level of 13 immune function pathways in each sample was quantified by ssGSEA. In addition, the TIP database precalculated the activities of the seven-step cancer-immunity cycle, and we obtained the scores for these steps to assess antitumor immunity [22]. Finally, the association between pyroptosis features and various immunomodulators was determined. The list of co-inhibitors, co-stimulators, HLA molecules, chemokines, interleukins, interferons and other cytokines was collected from previous publications (Table S4).

### Assessment of response to multiple therapeutics

We performed ROC curve analysis to predict RAI response based on the PyroScore in both the TCGA and GSE151179 cohorts. For immunotherapy, the score of tumor immune dysfunction and exclusion (TIDE) could predict the response to immune checkpoint inhibitors (ICIs) by estimating several published biomarkers, with a lower TIDE score indicating a better response to ICIs [23]. We also used the immunophenoscore (IPS) obtained from the Cancer Immunome Atlas (TCIA) to predict the response to anti-CTLA4 and anti-PD1 [24]. For molecular targeted therapy (MTT) and chemotherapy (CTx), the ‘oncoPredict’ package was used in conjunction with the Genomics of Drug Sensitivity in Cancer (GDSC) and Cancer Therapeutics Response Portal (CTRP) datasets for drug sensitivity analysis. The half-maximal inhibitory concentration (IC50) values for all drugs were predicted using a ridge regression model.

### External validation of PRGs in PTC

Six datasets containing information on PTC and NT samples were exported from the GEO database for external validation, including GSE29265 (20 PTC and 20 NT), GSE33630 (49 PTC and 45 NT), GSE60542 (33 PTC and 30 NT samples), conducted on the GPL570 platform, and GSE27155 (51 PTC and 4 NT samples), GSE5364 (35 PTC and 16 NT), GSE58545 (27 PTC and 18 NT), conducted on the GPL96 platform [25]. The batch effect between the different arrays was eliminated using the ComBat function of the ‘sva’ package. Protein expression of each candidate PRG in PTC and NT tissues was verified by immunohistochemical (IHC) staining from the HPA database [26]. The patient’s information and the antibodies used in IHC are listed in Table S5. In addition, gene expression profiles of 16 thyroid cancer cell lines were obtained through the CCLE database to verify the expression of PRGs at the cellular level [27].

### Quantitative real‑time polymerase chain reaction (qRT‑PCR)

With the approval of the Ethics Committee of China-Japan Union Hospital of Jilin University (No.20220804014), a total of 20 paired PTC and NT tissues were collected from the Thyroid Surgery Department of the China-Japan Union Hospital. Total RNA was extracted using RNAiso Plus (TaKaRa, Japan), followed by reverse transcription into cDNA using GoScript™ Reverse Transcription (Promega, USA). QRT- PCR was performed using GoTaq® qPCR Master Mix (Promega, USA). The reaction procedures were as follows: initiated by a 10 min incubation at 94 °C, followed by 40 cycles (95 °C, 15 s; 60 °C, 60 s). The mRNA levels were normalized to GAPDH as an internal control using the 2 − ΔΔCt method. The primers used in this study are listed in Table S6.

### Statistical analysis

All statistical analyses and graphical visualizations were carried out using R software and RStudio. The unpaired Student’s t test or the Mann‒Whitney U test was used to compare normally or nonnormally distributed variables. The Kruskal‒Wallis H test was used to compare several groups. The chi-square test was used to analyze the contingency table. Spearman analysis was used to determine the correlation between variables. A two-tailed *P* value < 0.05 was determined to indicate statistical significance.

## Results

### Three PyroCluster phenotypes were identified in PTC patients

**Fig. 1:**
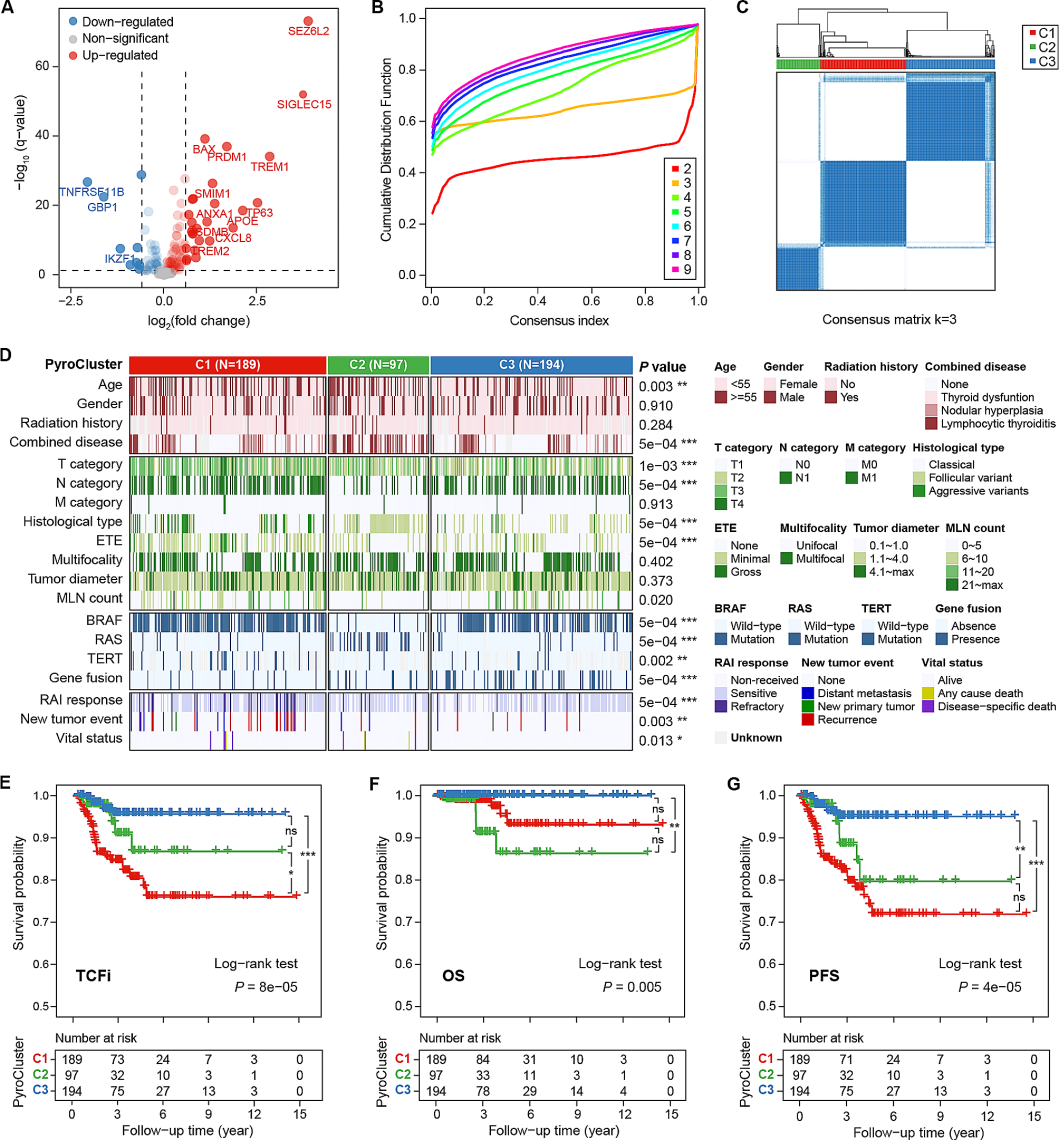
Expression profile, clinical relevance, and prognostic value of PRGs in PTC. **A** Volcano plot of 144 differentially expressed PRGs between PTC and normal thyroid tissues. **B** Cumulative distribution function of consensus clustering for k = 2 to 9. **C** Consensus clustering matrix shows that the 477 PTC patients from the TCGA dataset were classified into three PyroClusters (k = 3). **D** Differences in clinicopathologic, molecular, and prognostic features among PyroClusters. **E-G** The KM curves for TCFi (**E**), OS (**F**), and PFS (**G**) of PyroClusters.

Based on the results of the DEG analysis between PTC and NT tissues from the TCGA cohort, a total of 35 PRGs were identified as DEGs (fold change > 1.5, false discovery rate < 0.05); 26 were upregulated and 9 were downregulated (Fig. 1A). To investigate the relationship between the PRGs and the clinical subtypes of PTC, consensus clustering analysis was performed. As shown in Fig. 1B, the clustering variable (k) = 3 had the highest clustering stability from k = 1 to 9, indicating that PTC patients could be classified into three clusters (designated as C1, C2, and C3) with high intragroup and low intergroup correlations (Fig. 1C). The three PyroClusters exhibited significant differences in terms of clinicopathologic, molecular and prognostic features (Fig. 1D). C1 appeared to be more invasive, with a higher proportion of patients with advanced T and N category, aggressive histological variants and extrathyroidal extension (ETE), and molecular changes mainly involving BRAF and other gene mutations, resulting in a worse prognosis (more RAI-refractory and new tumor events). C2 was characterized by a follicular variant, RAS family mutations, more patients over 55 years of age with nodular hyperplasia, fewer cases of ETE and lymph node metastasis but also some deaths. C3 consisted mainly of younger patients with the classical histological subtype, BRAF mutation with gene fusion, and moderate tumor invasiveness but optimal prognosis.

To clarify the prognostic differences between the PyroClusters and to select the most representative endpoint indicators for PTC prognosis, we performed KM analysis for TCFi, PFS and OS. As shown in Fig. 1E-F, C1 presented the worst TCFi and PFS, while C2 showed the worst OS. The reason was few patients reached the endpoint for OS (death), which could lead to bias. In addition, due to interference from all-cause deaths and new tumor events, the difference in PFS between C1 and C2 was reduced. Overall, we proposed that TCFi was a more reliable and stable prognostic outcome for PTC than OS or PFS and that the three PyroClusters could effectively distinguish prognostic phenotypes.

### PyroScore model for predicting TCFi in PTC patients

**Fig. 2:**
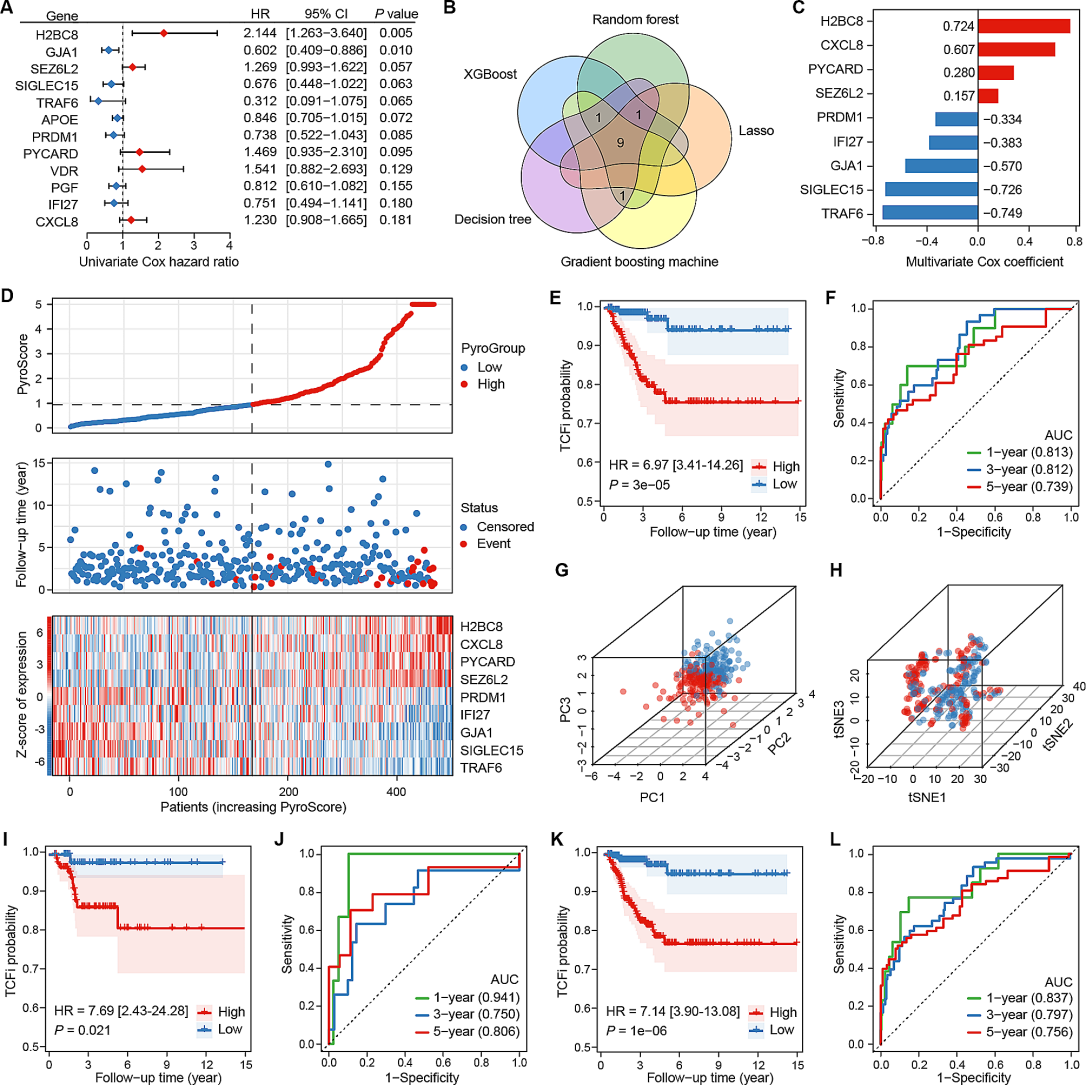
Construction and validation of a PyroScore prognostic model. **A** Univariate Cox regression analyses of PRGs associated with TCFi. The top genes with a *P* value < 0.2 are presented. **B** Venn diagram shows that five machine learning algorithms screened nine genes for constructing the prognostic model. **C** Coefficients of nine genes identified by multivariate Cox regression analyses. **D** PyroScore distribution, prognostic status of each patient, and heatmaps of nine prognostic PRGs. **E** TCFi KM curves of PyroScore subgroups. **F** ROC curves present the predictive efficiency of the PyroScore. **G, H** PCA plot **(G)** and t-SNE plot **(H)** show the separation of PyroScore subgroups. **I-L** TCFi KM curves and the ROC curves of PyroScore subgroups in the testing set **(I, J)** and the entire cohort **(K, L)**

To converge the PRGs for better quantification of the level of pyroptosis and to further demonstrate their predictive value for TCFi, the PyroScore model was constructed and validated. In the training set, 12 PRGs were preliminarily identified as associated with TCFi by univariate Cox regression analysis (Fig. 2A). Subsequently, five additional machine learning algorithms selected nine PRGs with the highest weights (TRAF6, SIGLEC15, H2BC8, CXCL8, GJA1, IFI27, PRDM1, PYCARD, SEZ6L2) for model construction (Fig. 2B). Multivariate Cox regression yielded the weight coefficients of these genes for the PyroScore calculation (Fig. 2C). According to the median PyroScore, patients were divided into high and low PyroScore subgroups (Fig. 2D). As illustrated in the KM analysis (Fig. 2E), the high PyroScore group had a significantly worse TCFi than the low PyroScore group (HR = 6.97, *P* = 2.3e-05). The ROC curves showed that the AUC values of the PyroScore for predicting 1-, 3-, and 5-year TCFi were 0.813, 0.812, and 0.739, respectively (Fig. 2F). Furthermore, the results of PCA and tSNE dimensionality reduction analysis corroborated that the grouping had good discriminatory power. The stable performance was further verified in both the testing set (Fig. 2I, J; Fig. S1A-C) and the entire cohort (Fig. 2K, L; Fig. S1D-F). The above results confirmed that the PyroScore model can effectively and robustly predict the TCFi of PTC patients.

### Development of an integrated nomogram to optimize the risk stratification of PTC

**Fig. 3:**
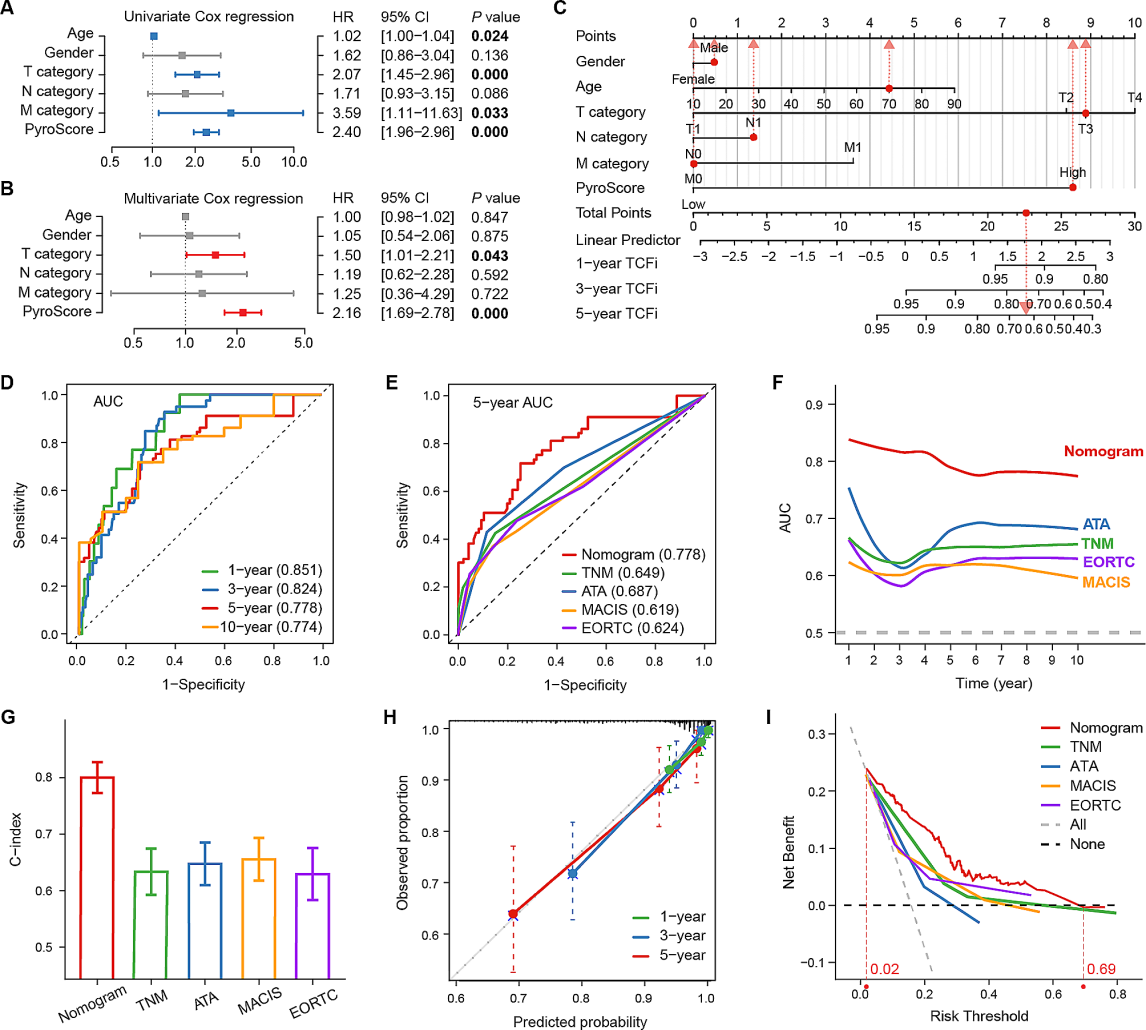
Development and evaluation of a nomogram integrating the PyroScore in the entire cohort. **A, B** Univariate **(A)** and multivariate **(B)** Cox regression analyses for validating the independent prognostic value of the PyroScore. **C** A nomogram to predict the 1-, 3-, and 5-year TCFi. The red dot shows the nomogram points of patients with barcode TCGA-DE-A4MD (recurrence in 267 days). **D** Time-dependent ROC curves of the nomogram to predict the 1-, 3-, 5-, and 10-year TCFi. **E** ROC curves of the nomogram and four THCA staging systems to predict the 5-year TCFi. **F** AUC for the nomogram and four staging systems based on the time-dependent ROC curves. **G** C-indexes of the nomogram and four staging systems. **H** Calibration curves of 1-, 3- and 5-year TCFi probability for the nomogram. **I** DCA curves of the nomogram and four staging systems for predicting the net benefit of the 5-year TCFi.

To determine the independent prognostic value of the PyroScore, univariate and multivariate Cox regression analyses were first performed in the entire cohort. PyroScore was positively and independently associated with TCFi in PTC (HR = 2.40, *P* < 0.001), and age and T and M categories were also related to TCFi (Fig. 3A, B). A nomogram integrating the PyroScore and clinicopathological features was then constructed to predict the TCFi (Fig. 3C). Time-dependent ROC curves showed that the nomogram had high AUCs for predicting the TCFi at the 1-, 3-, 5-, and 10-year time points (0.851, 0.824, 0.778, and 0.774, respectively) (Fig. 3D). We further compared the nomogram with the ATA, EORTC, MACIS, and TNM staging systems (Fig. 3E-F). The nomogram showed better discriminatory ability with higher time-dependent AUCs for 1- to 10-year TCFi and a good C-index for 5-year TCFi (Fig. 3G). The calibration curves depicted that the nomogram predicted the TCFi well at 1-, 3-, and 5-year (Fig. 3H). The 5-year DCA curves showed that the nomogram can achieve a greater net benefit than other risk stratification for TCFi prediction at a threshold probability of 0.02 to 0.69 (Fig. 3I). Overall, the PyroScore nomogram demonstrated considerable ability to improve prognostic risk prediction for PTC.

### Pathway enrichment levels vary among different pyroptosis patterns of PTC

**Fig. 4:**
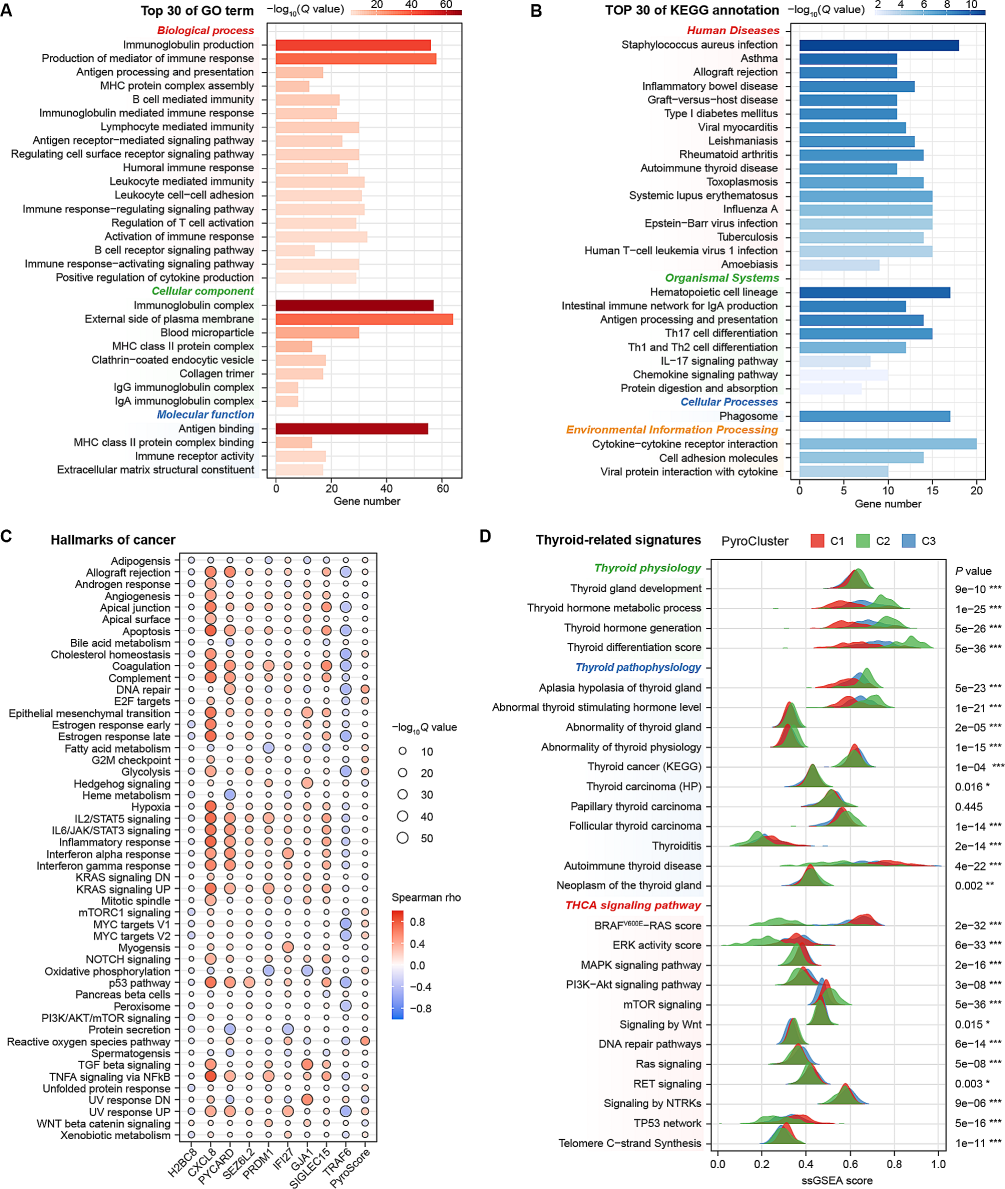
Signaling pathway enrichment analysis of the PRG signatures. **A, B** Top 30 of GO terms (**A**) and KEGG pathway annotations (**B**) enriched in upregulated DEGs between the PyroScore subgroups. **C** Heatmap shows the Pearson correlation between GSVA enrichment analysis scores of 50 hallmarks of cancer and PRG signatures. **D** The ridgeline chart shows the distribution differences in ssGSEA scores of THCA characteristic signatures in three PyroClusters (Kruskal–Wallis H test)

To elucidate the underlying signaling mechanisms that may be associated with pyroptosis patterns. GO and KEGG enrichment analyses were performed on the DEGs between the PyroScore subgroups. The upregulated DEGs in the high PyroScore group were mainly enriched in immune-related GO terms and KEGG pathways (Fig. 4A, B). The downregulated DEGs were enriched in various oncogenic signaling pathways (Fig. S2). Within the 50 cancer hallmarks, the PyroScore showed the strongest positive correlation with DNA repair, reactive oxygen species and the G2M checkpoint, while it was negatively associated with Hedgehog signaling, UV_Response_DN and KRAS signaling (all *P* < 0.001, Fig. 4C). Moreover, the differences in ssGSEA scores of 27 thyroid-related signatures among PyroClusters were further investigated (Fig. 4D). C2 exhibited RAS-like tumor characteristics, with a higher thyroid differentiation score (TDS), lower ERK and greater mTOR signaling pathway activation. C1 and C3 manifested BRAF-like tumor characteristics, with C1 having the lowest TDS and significant activation of thyroid autoimmune, MAPK, TP53 and TERT-related signaling pathways, while C3 showed activation of the PI3K-Akt and Ras signaling pathways. The above results suggest that pyroptosis may be involved in the development of PTC through regulating the immune response process.

### Effect of pyroptosis on TIME remodeling in PTC

**Fig. 5:**
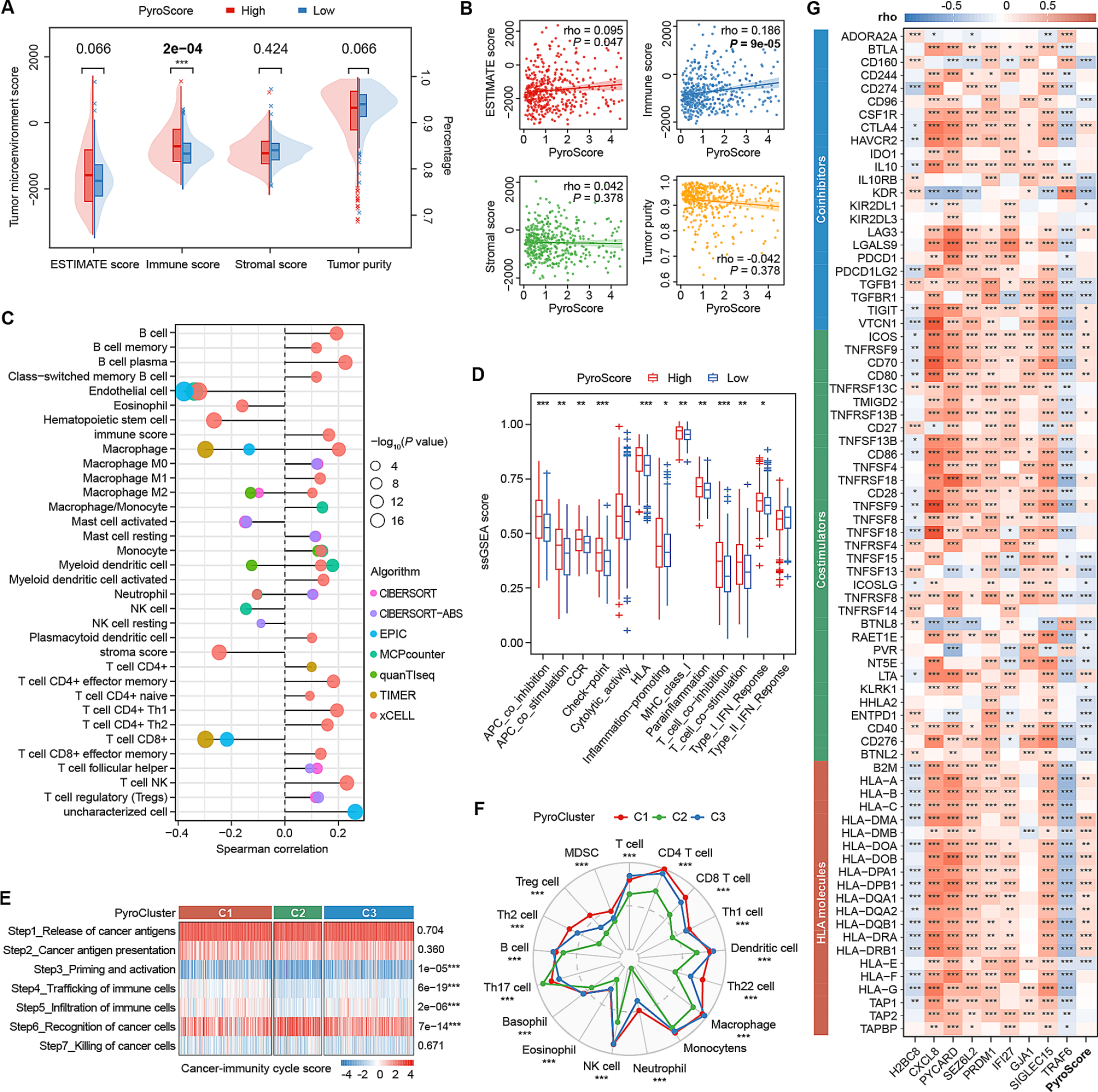
The immune landscape associated with pyroptosis in PTC. **A** Comparison of ESTIMATE scores between the PyroScore subgroups. **B** Association between the ESTIMATE scores and the PyroScore. **C** Lollipop plot shows the Spearman correlation between immune cell infiltration and the PyroScore. **D** Comparison of the ssGSEA enrichment scores of immune function pathways between the PyroScore subgroups. **E** Heatmap demonstrating the difference in the seven-step anticancer immunity cycle activity among the three PyroClusters. **F** Radar plot shows the difference in the ability of PyroClusters to recruit immune cells. **G** Spearman correlation between immunomodulators (co-inhibitors, co-stimulators and HLA molecules) and PRG signatures

To further elucidate the specific impact of pyroptosis on immune status, we performed comprehensive immune analyses. The ESTIMATE algorithm was used to check the components of the tumor microenvironment (Fig. 5A, B). The immune score was significantly higher in the high PyroScore group (*P* = 2e-04) and correlated positively with the PyroScore (rho = 0.186, *P* = 9e-05). However, there were no differences in stromal score or tumor purity between the PyroScore groups. Regarding the abundance of immune cells, the PyroScore was positively associated with most B cells, T cells, myeloid dendritic cells, monocytes, etc., and mainly negatively correlated with endothelial cells and CD8 + T cells (Fig. 5C). ssGSEA showed that several immune function pathways, such as checkpoint, HLA, and co-inhibition pathways, were highly enriched in the high PyroScore group (Fig. 5D).

Furthermore, we clarified the antitumor effect of immune cells by assessing the activity of seven steps in the anticancer immune cycle. C1 and C3 showed enhanced activity in step 3 (priming and activation), step 4 (trafficking of immune cells to tumor), and step 5 (infiltration of immune cells into tumor) and attenuation in step 6 (recognition of cancer cells) (Fig. 5E). In addition, we examined the recruitment of different immune cells in step 4 in more detail. As shown in Fig. 5F, BRAF-like tumors (C1 and C3) exhibit a stronger ability to recruit most immune cells than RAS-like tumors (C2). In particular, the recruitment activities for Treg cells, Th22 cells, neutrophils, Th2 cells and MDCs increased with the PyroScore. Moreover, a strong correlation between the PyroScore and model genes with several immunoregulatory molecules was observed (Fig. 5G; Fig. S3). These results revealed the critical role of pyroptosis in regulating the TIME and the potential application of immunotherapy.

### Pyroptosis features predicting the response to multiple treatments in PTC-RAI patients

**Fig. 6:**
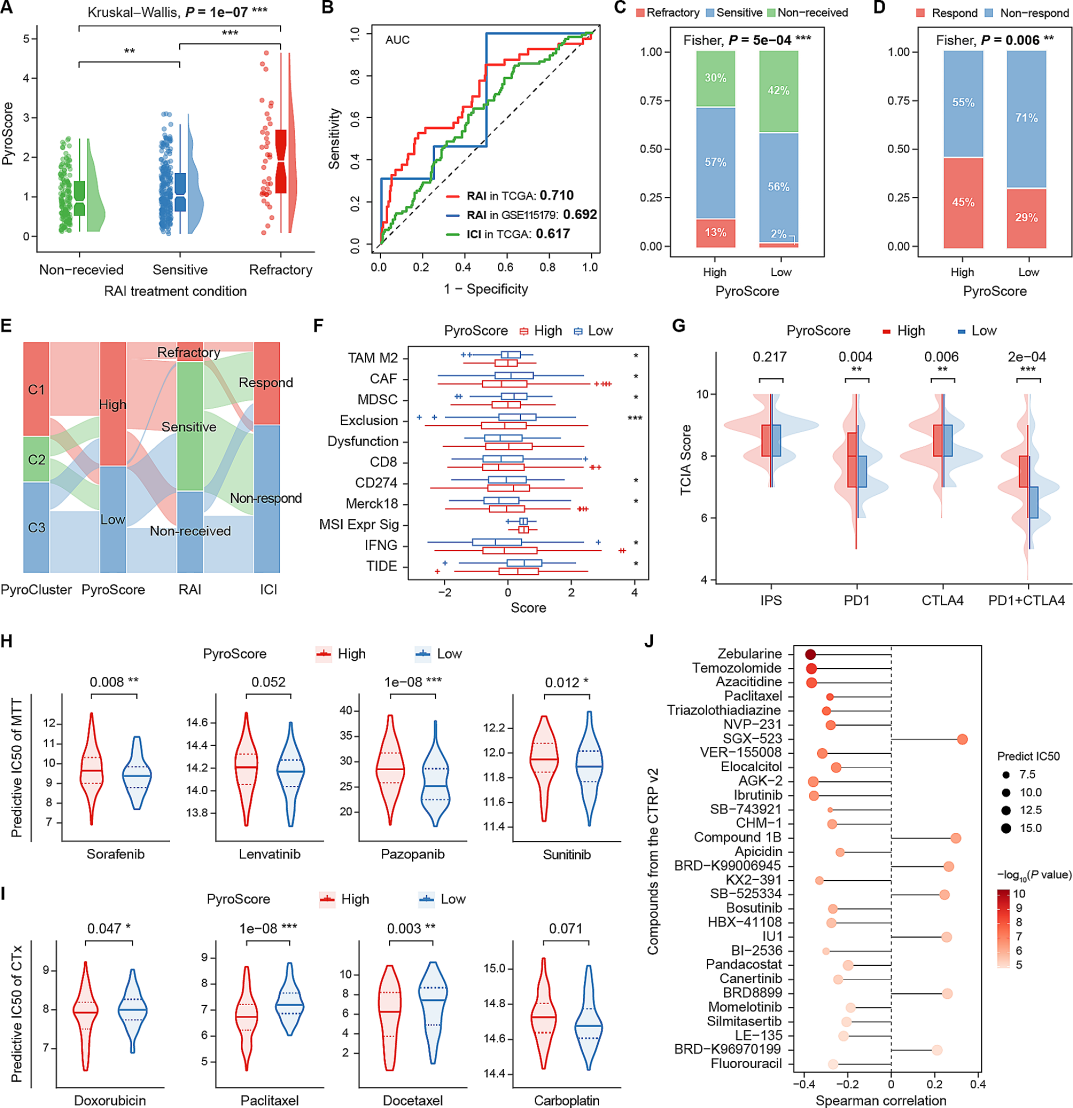
Prediction of multiple treatment responses based on pyroptosis features. **A** PyroScore increased dependently among the RAI treatment condition groups. **B** ROC curves of the PyroScore to predict the responsiveness of PTC-RAI patients to RAI and ICI treatments. **C, D** Distribution differences in objective RAI treatment responses (**C**) and putative ICI treatment responses derived from TIDE (**D**) between PyroScore subgroups. **E** Sankey diagram shows the case flow to various sources or sinks between two types of PRG signatures and RAI and ICI treatment responses. **F** Comparisons of the scores of TIDE, interferon-gamma (IFNG), microsatellite instability (MSI), Merck18 (T-cell-inflamed signature), CD274, CD8, T-cell dysfunction, T-cell exclusion, myeloid-derived suppressor cells (MDSCs), cancer-associated fibroblasts (CAFs), and M2 tumor-associated macrophages (TAMs) in PyroScore subgroups. **G** TCIA algorithm predicted the probability of the response to PD1/PDL1 and CTLA4 blockers in the PyroScore subgroups. **H, I** The IC50 of MTT **(H)** and CTx agents **(I)** between PyroScore subgroups. **J** Top 30 of compounds (from the CTRP v2. database) with the most significant differences in predict IC50 between PyroScore subgroups

Given the pivotal value of pyroptosis in the progression and prognosis of PTC, we further investigated whether pyroptosis features could help in determining treatment strategies. As systemic RAI is still the fundamental treatment for THCA, we compared the PyroScore under different RAI conditions. PyroScore increased dependently among the non-received, RS, and RR groups (*P* = 1e-07, Fig. 6A). The AUC for the PyroScore in predicting the response to RAI was 0.710 (Fig. 6B). The high PyroScore group had more PTC-RAI patients (70% vs. 58%) and a higher RR ratio (13% vs. 2%) than the low PyroScore group (*P* = 5e-04, Fig. 6C). The sankey plot also showed that most RR cases were from the high PyroScore group (Fig. 6E). Similarly, validation in the GSE151179 cohort confirmed the robust power of the PyroScore in discriminating RR and RS (AUC = 0.692, Fig. 6B).

RR patients have an unfavorable prognosis and require more effective therapies. However, there is limited data on these patients, so we performed the following analysis in patients of the PTC-RAI subset (*n* = 308), who had more severe disease and were more likely to be eligible for multiple therapies than patients without RAI treatment. For immunotherapy, the response to ICIs was initially predicted via the TIDE algorithm. The high PyroScore group had a lower TIDE score and more ICI responders (45% vs. 29%, *P* = 0.006, Fig. 6D, F), and most ICI responders were from the high PyroScore group (Fig. 6E). Besides, the high PyroScore group also had higher IFNG, Merck18 and CD274 scores, while the low PyroScore group showed T-cell exclusion characteristics and lower TAMM2, CAF and MDSC scores (Fig. 6F). As shown in Fig. 6G, patients with a high PyroScore presented a better response to CTLA4-/PD1+, CTLA4+/PD1-, CTLA4+/PD1 + blockers (all *P* < 0.01), indicating greater benefits from ICIs. For MTT, the low PyroScore group was more sensitive to pazopanib, sunitinib and sorafenib (Fig. 6H). For CTx, the high PyroScore group showed a better response to paclitaxel, docetaxel and doxorubicin (Fig. 6I). In addition, the high PyroScore patients benefitted more from zebularine, temozolomide and azacitidine (Fig. 6J). In brief, pyroptosis features are ideal predictors of the response to different treatments, which broadens the choice of new therapeutic strategies for PTC patients.

### Clinical, internal and external validation of PRGs in PTC

**Fig. 7:**
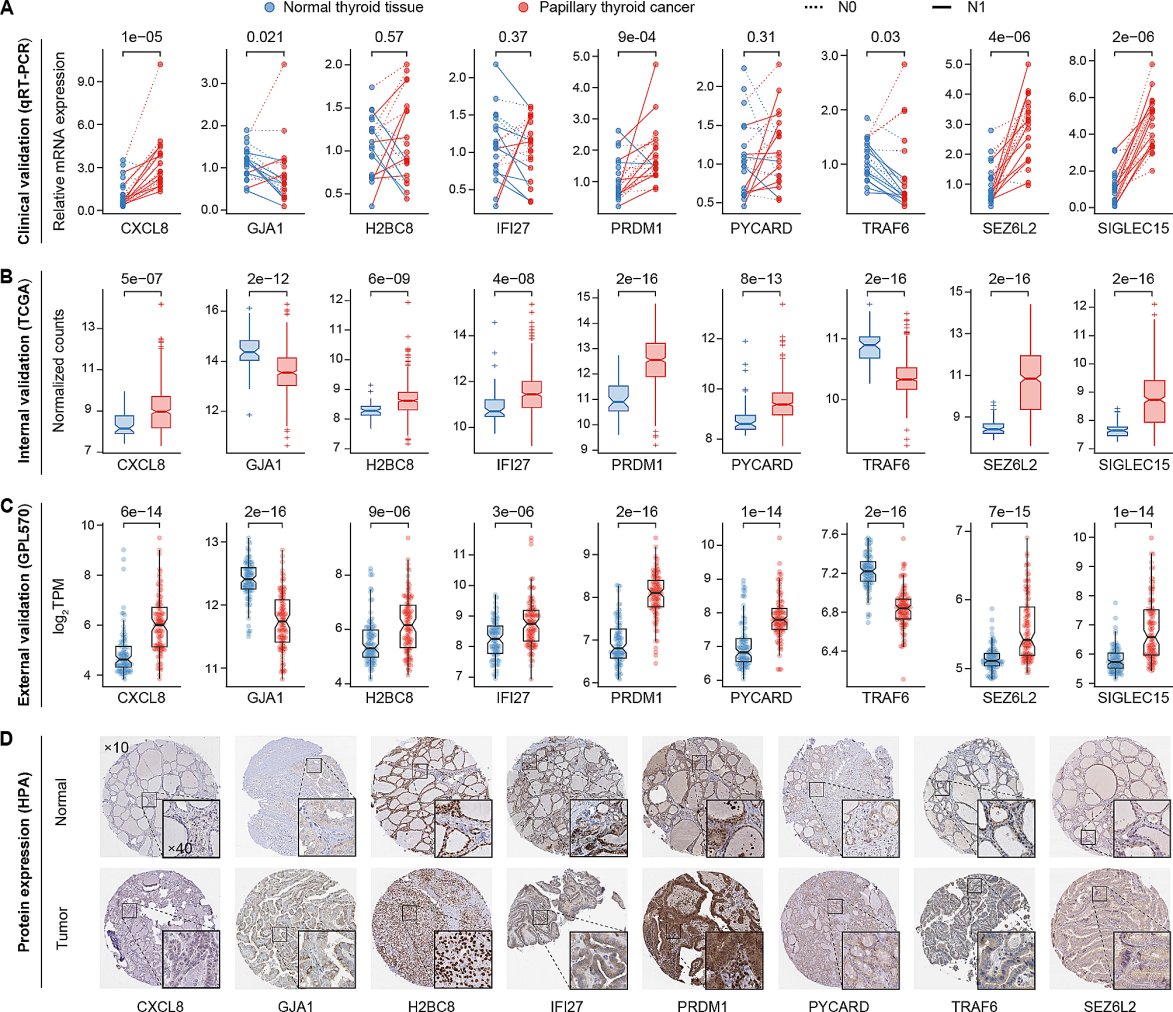
Clinical, internal and external validation of PRGs in PTC. **A** The mRNA expression levels of nine PRGs quantified by qRT‒PCR analysis in 20 pairs of clinical samples. **B** Internal validation of the mRNA expression of nine PRGs between 477 PTC and 59 NT from the entire TCGA cohort. **C** External validation of the mRNA expression of nine PRGs between 102 PTCs and 95 NTs from the GEO database (GPL570 sets). **D** Representative IHC staining images presenting the protein expression levels of nine PRGs in NT and PTC tissues from the HPA database. ×10: scale bar = 200 μm; ×40: scale bar = 50 μm

As the above analyses were all from public databases, we collected further clinical samples to validate them in the real world. The expression of nine prognostic PRGs was validated by qRT‒PCR in 20 pairs of PTC and NT clinical samples. As shown in Fig. 7A, CXCL8 (*P* = 1e-05), PRDM1 (*P* = 9e-04), SEZ6L2 (*P* = 4e-06) and SIGLEC15 (*P* = 2e-06) were significantly upregulated, while GJA1 (*P* = 0.021) and TRAF6 (*P* = 0.03) were downregulated in PTC tissue. The comparative results were consistent with the results of the bioinformatics analysis. There were no significant differences in H2BC2, IFI27 and PYCARD; however, the trend was still observed in N1 patients, by expansion of the sample size may be required to provide further evidence.

Furthermore, we performed internal validation in the entire TCGA cohort (Fig. 7B) and external validation in two GEO collections, namely, the GPL60 sets (Fig. 7C) and the GPL570 sets (Fig. S4). All genes showed marked differences between PTC and NT tissues, and the trend was consistent with previous results. Additionally, these genes exhibit varying levels of expression in THCA cell lines, with SEZ6L2 showing higher expression levels in cell types of increased malignancy (Fig. S5). In addition, the protein levels of these nine genes were examined in more detail. As already observed at the mRNA expression level, the IHC results showed that CXCL8, H2BC8, IFI27, PRDM1, PYCARD and SEZ6L2 proteins were obviously highly expressed in PTC tissues, while GJA1 and TRAF6 proteins were downregulated (Fig. 7D).

## Discussion

**Fig. 8:**
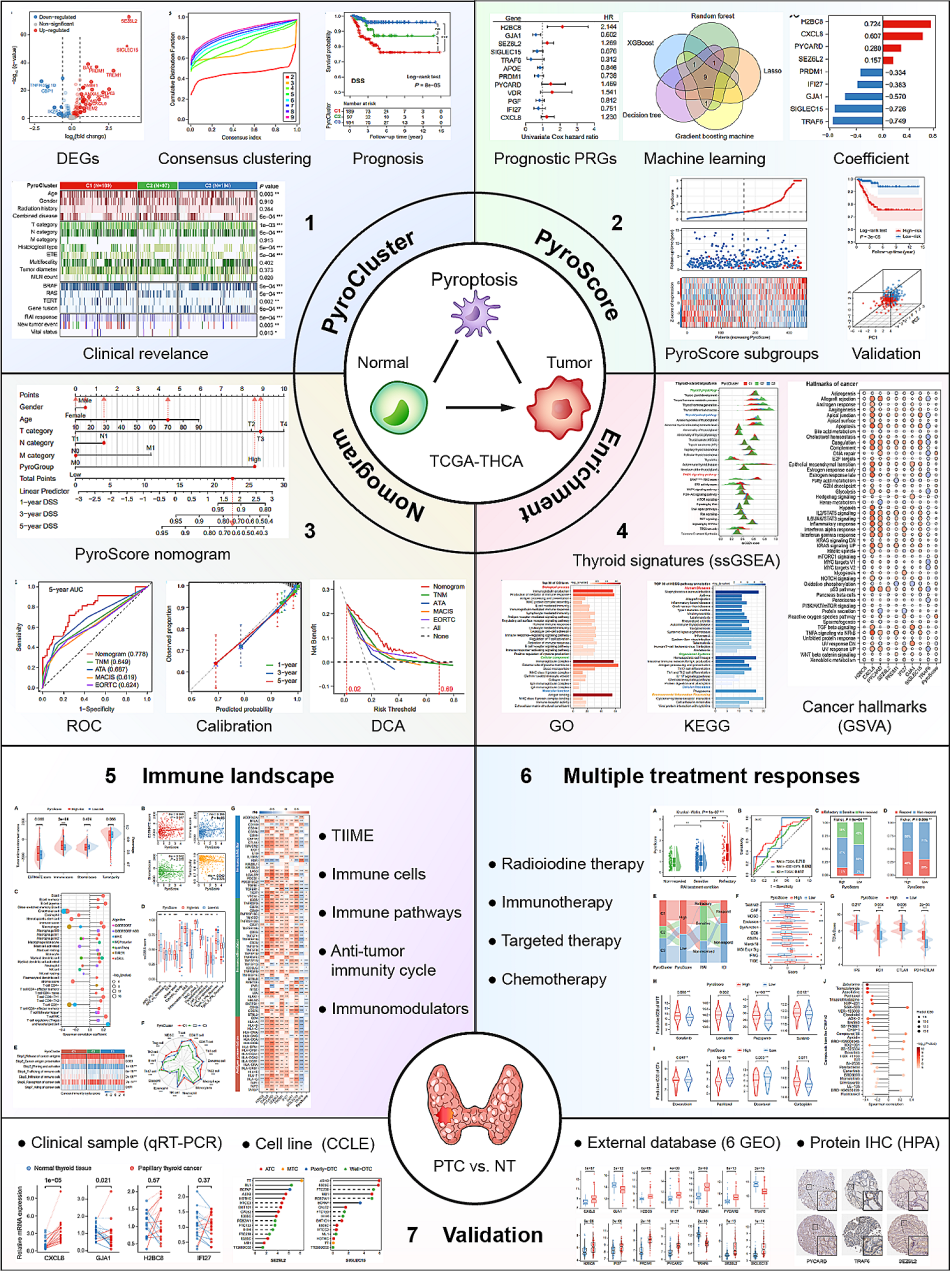
Flow chart of the present study

Our study comprehensively investigated the role of pyroptosis in the pathogenesis and prognosis evaluation of PTC (Fig. 8). We grouped PTC patients into three PyroClusters and constructed the PyroScore model to predict prognosis. Notably, we focused on the prognostic indicator TCFi, which is more short-term, accurate, and valuable for PTC prognosis prediction than OS or PFS. We also thoroughly investigated the association between pyroptosis and TIME remodeling in PTC and confirmed the value of pyroptosis features in decision-making regarding the application of multimodal treatment strategies, expanding insights into future therapeutic approaches for PTC. We verified the expression of nine PRGs in PTC and NT tissues in six GEO datasets, as well as by qRT‒PCR in 20 clinical samples, and obtained roughly consistent results as the previous bioinformatics analysis.

Pyroptosis is a highly inflammatory programmed cell death that can trigger a cascade of inflammatory responses and disrupt the homeostasis of the TIME. It is involved in many inflammatory diseases, tumors and immunotherapy effects [4, 6, 8]. The dual role of pyroptosis in pathogenesis is similar to the effect of chronic inflammation in thyroid diseases. Some studies have confirmed that excessive iodine induces the activation of inflammasomes and pyroptosis in thyroid follicular epithelial cells, which is associated with the occurrence of autoimmune thyroiditis [28–30]. Cadmium exposure could promote pyroptosis of thyroid follicular cells, thereby disrupting thyroid tissue structure and endocrine function [31]. Lidocaine may ameliorate subacute thyroiditis by inhibiting the pyroptosis pathway and curbing the expression of inflammatory factors [32]. Wu et al. constructed a risk model to predict the OS of THCA patients based on 31 PRGs. The model showed a significant increase in tumor immune cell infiltration in the low-risk group. Zhao et al. identified a novel antitumor effect of pyroptosis induced by the combination of apatinib and melittin in anaplastic THCA [33]. The distinct effects of pyroptosis in different thyroid diseases reflect complex molecular regulatory mechanisms. However, studies on the functions and mechanisms of pyroptosis in THCA remain limited, and there are no studies on this topic in PTC. Therefore, our study focused on exploring the impact of pyroptosis on the clinical phenotype of PTC and its value for predicting prognosis and the response to various treatments.

In this study, PTC patients were classified into three PyroClusters based on the differentially expressed PRGs between PTC and NT. According to the results of the clinical relevance analysis and functional enrichment analysis, we concluded that C1 and C3 were BRFA-like tumors and C2 was a RAS-like tumor (Figs. 1D and 4D). It is known that differentiated THCAs develop from follicular cells and follow two distinct direction: BRAF-like and RAS-like [34]. Currently, BRAF mutation tests are widely used in clinical practice to facilitate the diagnosis of THCA. However, BRAF status alone is not sufficient to contribute substantially to risk stratification in most patients [17]. The results showed that C1 had a markedly worse prognosis than C3, even though both clusters represented BRFA-like tumors. Although some C1 cases had additional mutations and C2 had the most cases of gene fusion, these features could not fully explain the significant differences between the two clusters of patients overall. Thus, we speculated that pyroptosis exerts some influence on the evolution of THCA, as some studies have confirmed that pyroptosis is also involved in the regulation of MAPK, ERK and PI3K/Akt signaling pathways, which are important signaling pathways related to the pathogenesis of THCA [35, 36]. Our study first revealed the considerable ability of pyroptosis features to distinguish molecular subtypes of PTC, which is expected to be applied to support precision diagnosis and risk stratification.

Before further constructing the prognostic model based on the PRGs, we clarified the value of different time-to-event endpoints in the prognosis assessment of PTC. Many prognostic models choose OS as an endpoint, but PTCs are mostly indolent tumors with few deaths, making OS of limited value for early clinical guidance. PFS is considered more appropriate for THCA than OS [14], but the PFS events provided by the TCGA database included new tumors and all-cause deaths; these events were not closely associated with PTC, and their inclusion could cause statistical bias. As shown in Fig. 1G, there was no difference in PFS between C1 and C2. Therefore, referring to the DATECAN initiative (Definition for the Assessment of Time-to-event Endpoints in CANcer trials) [16], we propose to select TCFi as the clinical endpoint for PTC, as it considers events of recurrence, metastasis and specific death from THCA. TCFi could capture more events during follow-up and better reflect the prognosis associated with PTC specificity. Visually, among the potential prognostic indicators, TCFi presented the most significant differences in the KM curves of the PyroClusters (Fig. 1E).

Next, we constructed a PyroScore signature consisting of nine PRGs, and then integrated the PyroScore and clinical features to develop a nomogram model that had better discrimination and net benefit than the ATA, TNM, MACIS and EORTC staging systems. Of the nine model PRGs, four (H2BC8, CXCL8, PYCARD, and SEZ6L2) were upregulated in PTC and related to a poor prognosis; two (GJA1 and TRAF6) were downregulated and were favorable prognosis factors, implying that they have a consistent effect on tumorigenesis and progression of PTC, and can be considered as oncogenes and tumor suppressors, respectively. Additionally, three genes (PRDM1, IFI27 and SIGLEC15) were highly expressed in tumor tissues but were also favorable prognostic factors, suggesting a paradox: they promote carcinogenesis but inhibit tumor progression. PRDM1 regulates all lymphocyte lineage cells and induces exhaustion modules in antigen-specific T cells [37]. It is expressed in almost all cases of Hashimoto’s thyroiditis and is involved in lymphoid tissue formation [38]. Hou et al. found that THCA patients with high SIGLEC15 expression manifested immune exhaustion, and immune cells in particular (especially monocytes and macrophages) express SIGLEC15 and thus could induce an immune escape environment [39]. IFI27 participates in innate immunity and apoptosis processes, reflecting an increase in immunopathology (either local or systemic). It has recently been identified as a biomarker for the early diagnosis and outcomes of COVID-19 that is involved in preexisting memory T-cell responses [40, 41]. Adam et al. noted that IFI27 could distinguish between different types of ICI-associated renal problems, as these entities have a high degree of inflammatory molecular overlap [42]. Hence, we hypothesize that these three genes may participate in chronic inflammation of the thyroid gland mediated by immunosuppression and T-cell exhaustion. Overall, the dysregulation of pyroptosis might play divergent roles at different stages of THCA.

Furthermore, in-depth functional analysis revealed significant enrichment of various signaling pathways associated with the immune response. To elucidate the intricate relationship between pyroptosis and immune regulation, we initially assessed the overall differences in the TIME across pyroptosis subgroups. Notably, the high PyroScore group exhibited a higher immune score, while there were no discernible differences in terms of tumor purity and stroma score (Fig. 5A). This finding confirms the specificity and strong correlation of pyroptosis in regulating the TIME. Regarding the infiltration of immune cells, we observed an increased abundance of phagocytic immune cells (such as myeloid dendritic cells, monocytes, and M0/M1 macrophages) as pyroptosis intensified. This phenomenon could be linked to the heightened production of reactive oxygen species and consequential DNA damage [43], consistent with our GSVA results (Fig. 4C). In addition, the infiltration of cytotoxic CD8 + T cells showed a decreasing trend, potentially leading to a diminished capacity for killing tumors [44]. These dual factors may underlie the poorer prognosis observed in patients with a high PyroScore. Remarkably, we also uncovered substantial activation of checkpoint, HLA, and co-inhibition pathways in the high PyroScore group. This strong association between the PyroScore and various immunomodulators further solidifies these findings.

A broader understanding of the interactions between PTC progression and the TIME holds the potential to usher in novel therapeutic approaches for aggressive PTC. Most PTC patients experience favorable outcomes following standard surgical procedures, TSH suppression and RAI therapy, but approximately 20%~30% exhibit resistance to RAI and/or recurrent events, and the 10-year OS rate remains below 10% [17, 45, 46]. Our PyroScore has demonstrated its ability to discern the response of PTC patients to RAI treatment in both the TCGA and GSE151179 cohorts, with respective AUC values of 0.710 and 0.692. Notably, the high PyroScore group exhibited a higher proportion of RR patients. Several studies have highlighted the potential of inducing and activating pyroptosis as a means to transform ‘cold’ tumors into ‘hot’ ones, thereby enhancing their responsiveness to immunotherapy [47, 48]. In our study, patients with a high PyroScore were found to have more antitumor lymphocytes, indicative of a ‘hot’ tumor phenotype, whereas those with a low PyroScore demonstrated a significantly higher degree of immune exclusion, characteristic of ‘cold’ tumors. The results derived from the TIDE and TCIA algorithms further confirm that the high PyroScore group is more likely to benefit from ICIs, including PD1/PDL1 and CTLA4 blockade. Numerous ongoing clinical trials are exploring the efficacy of MTT for THCA patients [49]. Our findings indicate that currently approved targeted drugs for THCA, such as sorafenib and pazopanib, offer greater benefits to patients with a low PyroScore, providing valuable insights for drug selection. Additionally, we identified potentially effective agents, such as ibrutinib and bosutinib, which could exhibit increased sensitivity in patients with high PyroScore. It is widely recognized that PTC is insensitive to CTx [17]. Our study assessed the responsiveness of PTC to four common CTx agents and found that patients with a high PyroScore tend to derive greater benefits, particularly from paclitaxel. Zhang et al. have previously demonstrated paclitaxel’s capacity to limit tumor proliferation and metastasis by inducing pyroptosis [50]. We also presented some new potential options for CTx agents, namely, zebularine, temozolomide, and azacitidine, which are linked to DNA methylation, synthesis and repair processes. Niu et al. inhibited tumor proliferation by inducing cancer cell pyroptosis with DNA methylation inhibitors [42]. Further foundational research will be necessary to validate their applicability in PTC treatment.

There are several limitations in the present study. Our predictive model lacks external validation, as currently, only the TCGA-THCA dataset contains prognosis information for PTC. Although we conducted internal validation by randomly splitting samples, it is still necessary to verify the model’s reliability with multicenter cohorts that includes larger sample size and adequate follow-up period. Meanwhile, further experimental studies are expected to elucidate the underlying mechanisms of PRGs and potential drugs in PTC. In addition, given the association between pyroptotic tumor cells and immune cells, we will explore their crosstalk effects through single-cell sequencing in future research.

## Conclusion

In summary, our study demonstrated that a novel nine PRGs signature has predictive value for the TCFi (includes recurrence, metastasis and specific death) of PTC. Pyroptosis exerts a significant influence in the remodeling of the TIME in PTC, and its derived features could predict the response to RAI in aggressive patients, and assist in decision-making regarding ICIs, MTT and CTx. These findings elucidate the immunological role of pyroptosis in the development of PTC and highlight its potential as prognostic biomarkers and therapeutic targets in the management of PTC patients.

### Electronic supplementary material

Below is the link to the electronic supplementary material.

Supplementary Material 1: Collection of supplementary tables. Table S1. Pyroptosis-related genes list. Table S2. Clinical characteristics in the training, the testing, and the entire sets from the TCGA database. Table S3. The gene sets of thyroid-related signatures. Table S4. Immunomodulatory genes list. Table S5. Detail information of the HPA patients and the antibodies. Table S6. Premier sequences for qRT-PCR analysis.

Supplementary Material 2: Collection of supplementary figures. Figure S1. Validating the PyroScore model in the testing set and the entire cohort. Figure S2. Enrichment analyses of the DEGs downregulated between the PyroScore subgroups. Figure S3. Spearman correlation between pyroptosis-derived signatures and chemokines, interferons, interleukins, and other cytokines. Figure S4. External validation of expression of nine prognostic PRGs in GPL96 sets. Figure S5. Expression levels of nine prognostic PRGs in thyroid cancer cell lines.

## Data Availability

No datasets were generated or analysed during the current study.

## Abbreviations

THCA: Thyroid cancer
PTC: Papillary thyroid cancer
TIME: Tumor immune microenvironment
NT: Normal thyroid
OS: Overall survival
PFS: Progression-free survival
TCFi: Thyroid cancer-free interval
RAI: Radioactive iodine
ATA: American Thyroid Association
RS: RAI-sensitive
RS: RAI-refractory
DEGs: Differentially expressed genes
LASSO: Least absolute shrinkage and selection operator
XGBoost: Extreme gradient boosting
KM: Kaplan–Meier
ROC: Receiver operating characteristic
PCA: Principal component analysis
t-SNE: t-distributed stochastic neighbor embedding
c-index: Concordance index
DCA: Decision curve analysis
MACIS: Metastases, Age, Completeness of Resection, Invasion, Size
TNM: Tumor, Node, Metastases
EORTC: European Organization for Research and Treatment of Cancer
GO: Gene Ontology
KEGG: Kyoto Encyclopedia of Genes and Genomes
GSVA: Gene set variation analysis
ssGSEA: Single sample gene set enrichment analysis
TIP: Tracking Tumor Immunophenotype database
TIDE: Tumor Immune Dysfunction and Exclusion database
ICIs: Immune checkpoint inhibitors
IPS: Immunophenoscore
TCIA: Cancer Immunome Atlas
MTT: Molecular targeted therapy
CTx: Chemotherapy
GDSC: Genomics of Drug Sensitivity in Cancer
CTRP: Cancer Therapeutics Response Portal
IC50: Half-maximal inhibitory concentration
IHC: Immunohistochemical
HPA: Human Protein Atlas database
qRT‑PCR: Quantitative real‑time polymerase chain reaction
ETE: Extrathyroidal extension
TDS: Thyroid differentiation score
