# Supplementary material for: Elucidating the role of Pyroptosis in papillary thyroid cancer: prognostic, immunological, and therapeutic perspectives
Source: Cancer Cell Int. 2024 Jan 29;24:45. doi: 10.1186/s12935-024-03229-0 (PMC10823616; doi:10.1186/s12935-024-03229-0)
Supplement: Supplementary file 2 — Supplementary Material 2: Collection of supplementary figures. Figure S1. Validating the PyroScore model in the testing set and the entire cohort. Figure S2. Enrichment analyses of the DEGs downregulated between the PyroScore subgroups. Figure S3. Spearman correlation between pyroptosis-derived signatures and chemokines, interferons, interleukins, and other cytokines. Figure S4. External validation of expression of nine prognostic PRGs in GPL96 sets. Figure S5. Expression levels of nine prognostic PRGs in thyroid cancer cell lines. [file 12935_2024_3229_MOESM2_ESM.docx]

**Electronic Supplemental Material 2**


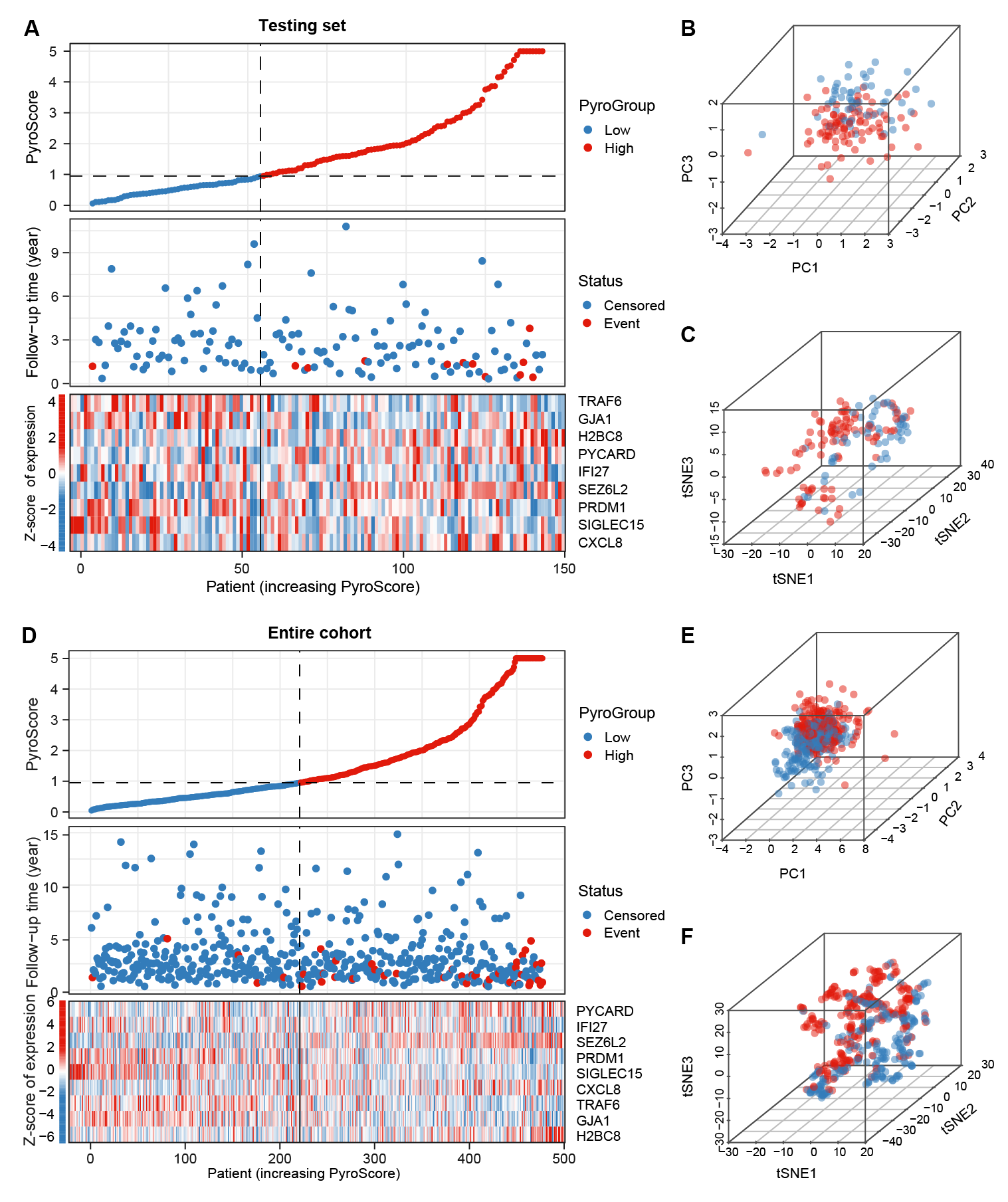


**Figure S1.** Validating the PyroScore model in the testing set and the entire cohort.

**(A, D)** PyroScore distribution, prognostic status of each patient, and heatmaps of nine prognostic PRGs. PCA plot (**B, C**) and t-SNE plot (**E, F**) show the separation of PyroScore subgroups.
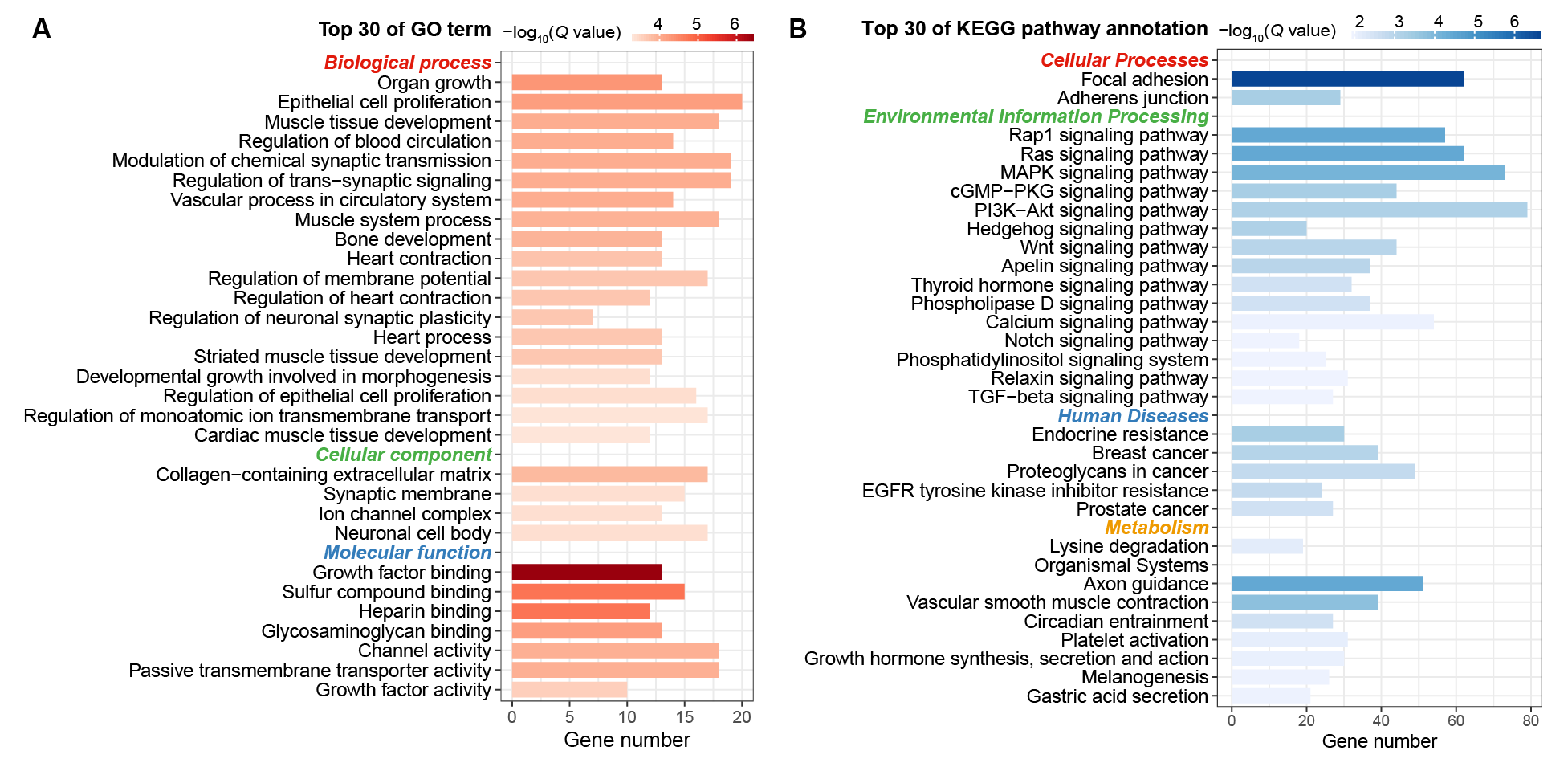


**Figure S2**. Enrichment analyses of the DEGs downregulated between the PyroScore subgroups. (**A)** GO term. **(B**) KEGG pathway annotation.


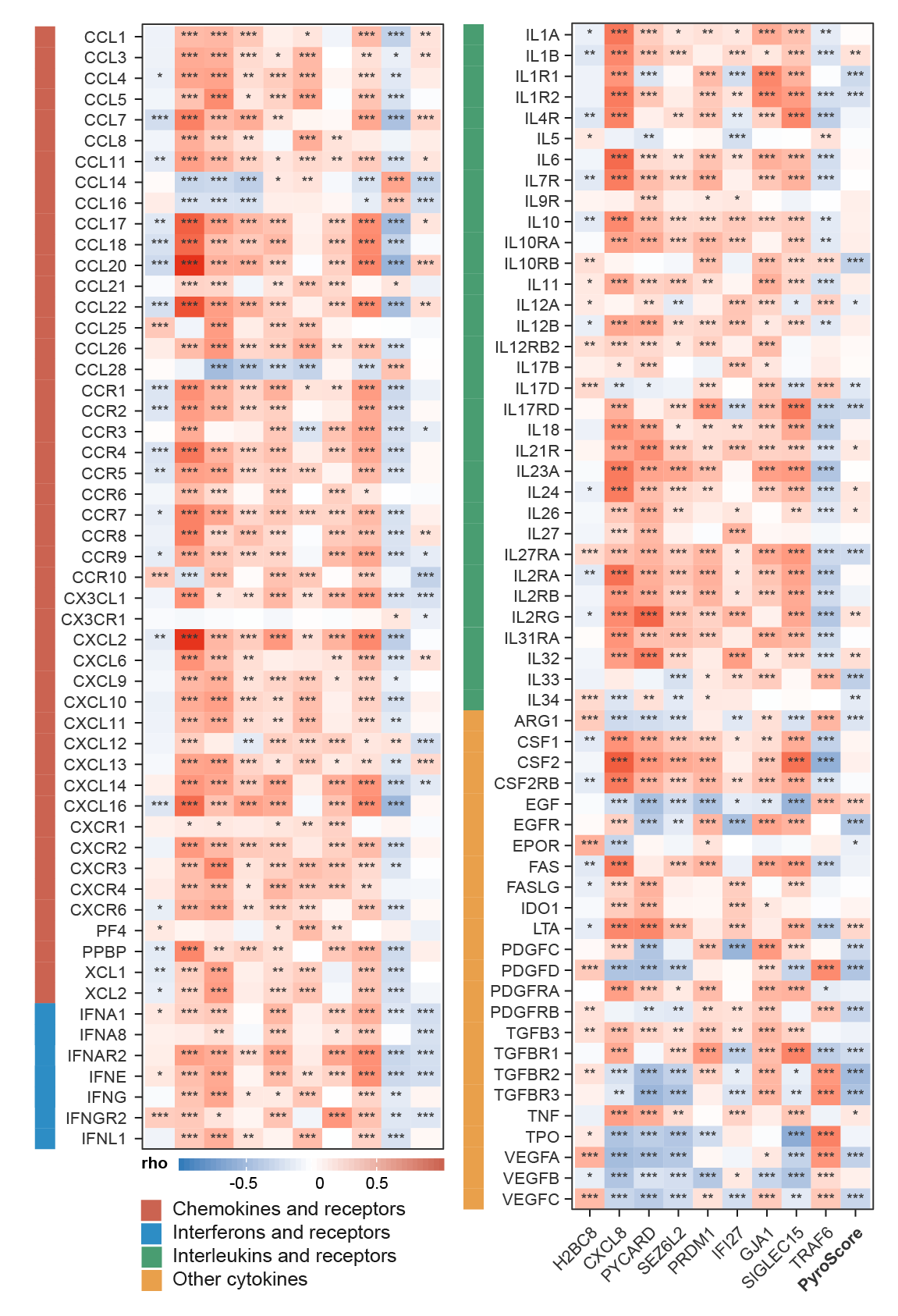


**Figure S3**. Spearman correlation between pyroptosis-derived signatures and chemokines, interferons, interleukins, and other cytokines.
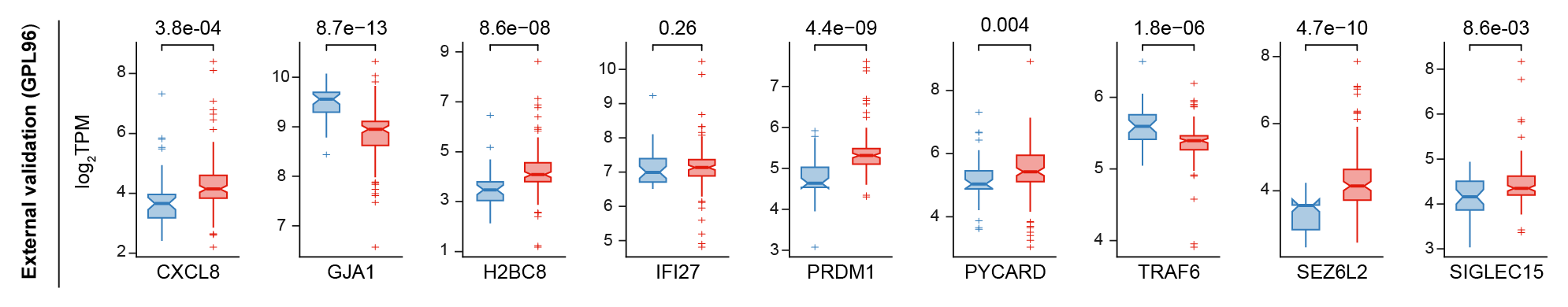


**Figure S4.** External validation of expression of nine prognostic PRGs in GPL96 sets.


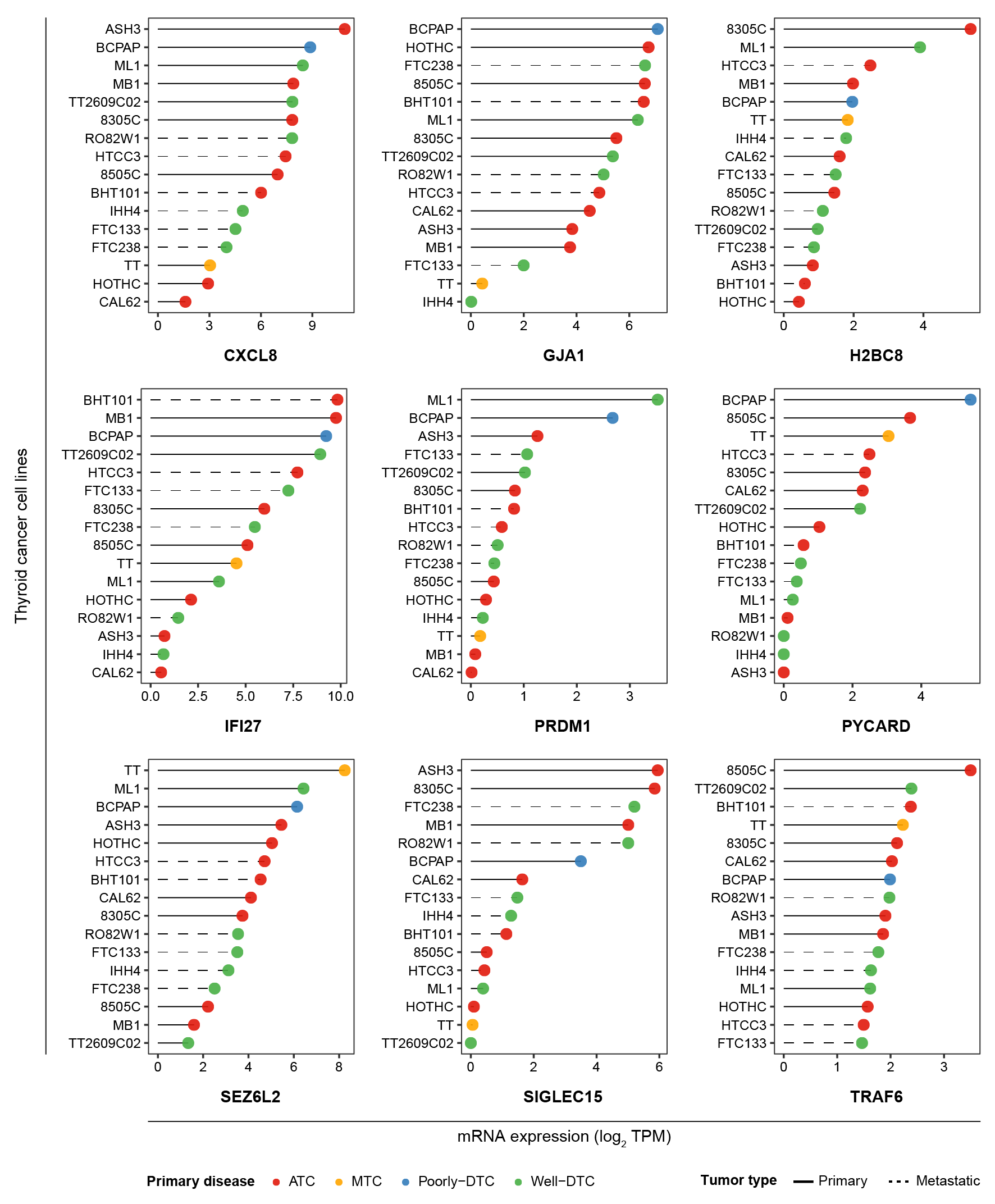


**Figure S5.** Expression levels of nine prognostic PRGs in thyroid cancer cell lines.
